# Consumption of kiwifruit capsules increases *Faecalibacterium prausnitzii* abundance in functionally constipated individuals: a randomised controlled human trial

**DOI:** 10.1017/jns.2017.52

**Published:** 2017-10-12

**Authors:** Paul Blatchford, Halina Stoklosinski, Sarah Eady, Alison Wallace, Christine Butts, Richard Gearry, Glenn Gibson, Juliet Ansell

**Affiliations:** 1 The New Zealand Institute for Plant & Food Research Limited, Private Bag 11600, Palmerston North 4442, New Zealand; 2 The New Zealand Institute for Plant & Food Research Limited, Private Bag 4704, Lincoln 8140, New Zealand; 3 Department of Medicine, University of Otago, Christchurch, New Zealand; 4 Department of Food and Nutritional Sciences, University of Reading, Reading RG6 6AP, UK

**Keywords:** Kiwifruit, Gut microbiota, Human studies, Constipation, *Faecalibacterium prausnitzii*, FC, functionally constipated, H, high dose, L, low dose

## Abstract

This study investigated the impact of ACTAZIN™ green (2400 and 600 mg) and Livaux™ (2400 mg) gold kiwifruit supplements on faecal microbial composition and metabolites in healthy and functionally constipated (FC) participants. The participants were recruited into the healthy group (*n* 20; one of whom did not complete the study) and the FC group (*n* 9), each of whom consumed all the treatments and a placebo (isomalt) for 4 weeks in a randomised cross-over design interspersed with 2-week washout periods. Modification of faecal microbiota composition and metabolism was determined by 16S rRNA gene sequencing and GC, and colonic pH was calculated using SmartPill^®^ wireless motility capsules. A total of thirty-two taxa were measured at greater than 1 % abundance in at least one sample, ten of which differed significantly between the baseline healthy and FC groups. Specifically, Bacteroidales and *Roseburia* spp. were significantly more abundant (*P* < 0·05) in the healthy group and taxa including Ruminococcaceae, *Dorea* spp. and *Akkermansia* spp. were significantly more abundant (*P* < 0·05) in the FC group. In the FC group, *Faecalibacterium prausnitzii* abundance significantly increased (*P* = 0·024) from 3·4 to 7·0 % following Livaux™ supplementation, with eight of the nine participants showing a net increase. Lower proportions of *F. prausnitzii* are often associated with gastrointestinal disorders. The discovery that Livaux™ supplementation increased *F. prausnitzii* abundance offers a potential strategy for improving gut microbiota composition, as *F. prausnitzii* is a butyrate producer and has also been shown to exert anti-inflammatory effects in many studies.

The gastrointestinal tract harbours approximately 10^14^ microbial cells, consisting of over 1000 species or phylotypes, the majority of which reside in the colon^(^^1^^,^^2^^)^. The colonic microbiota has been associated with a wide range of health benefits including improved immune function and maturation, modified behaviour, regulation of satiety, inhibition of pathogens and augmentation of mineral absorption^(^^3^^–^^5^^)^. Imbalances in microbial community composition may be caused by diet, genetics, age, stress or xenobiotics and can induce a state of dysbiosis that may promote a more disease-susceptible microbiota. Consumption of certain types of dietary components can have a major influence on the communities of colonic micro-organisms, with changes typically being observed within 24 h of consumption^(^^6^^,^^7^^)^.

Kiwifruit has been implicated in promoting several aspects of gut health including improving stool frequency, increasing commensal bacteria and improving aspects of immune function^(^^8^^–^^10^^)^. These benefits are thought to derive from the inherent levels of digestion-resistant carbohydrates, polyphenols and vitamin C^(^^11^^)^. The laxative effect of kiwifruit is undoubtedly the most well-studied health impact, whereas prebiotic effects are less well understood. In addition to host absorption of nutrients, enhancement of colonic microbial composition is thought to mediate many of the beneficial health effects of kiwifruit. Organic acids such as acetate, propionate and butyrate are produced as a result of microbial fermentation of kiwifruit digestion-resistant carbohydrates^(^^9^^)^. Organic acids may be absorbed by colonocytes into the bloodstream where they are further metabolised in the liver, muscle and other peripheral tissues, and are associated with the mediation of immune effects, cholesterol, satiety, and improved mineral absorption^(^^12^^–^^14^^)^.

As the importance of a robust and diverse colonic microbiota is increasingly apparent and the potential of kiwifruit to modulate the microbiota is being unearthed, a human intervention study was conducted to ascertain the effect of kiwifruit-derived supplements on colonic microbial composition and metabolism. Two kiwifruit-derived supplements, ACTAZIN™ green-fleshed (*Actinidia chinensis* var. *deliciosa* ‘Hayward’) and Livaux™ gold-fleshed (*Actinidia chinensis* var. *chinensis* ‘Zesy002’) (Anagenix Ltd), were used as dietary interventions in the trial. These capsules are cold-processed dietary supplements, from which the skin and seeds are removed, formulated to maintain the integrity of innate kiwifruit compounds. The objective of this study was to assess the impact of ACTAZIN™ and Livaux™ on the composition of colonic bacteria, metabolites and gut pH, which was measured by 16S rRNA gene sequencing, organic acid analysis and SmartPill^®^ technology, respectively.

## Materials and methods

### Study protocol

The study design was a randomised double-blind placebo-controlled cross-over trial with participants consuming four different interventions for 4 weeks each, with a 2-week washout between each intervention. A schematic view of the trial design is shown in Supplementary Fig. S1. The interventions were delivered in 4 × 600 mg capsules supplied by Anagenix Ltd prepared to look identical to preserve intervention blinding (Table 1). Detailed information on ingredient composition, trial recruitment and participant inclusion and exclusion criteria can be found in the Ansell *et al.*^(^^15^^)^ paper reporting laxation endpoints of this trial. Briefly, participants consumed four different intervention combinations: placebo (isomalt coloured green) (2400 mg/d), ACTAZIN™ L (600 mg/d), ACTAZIN™ H (2400 mg/d) and Livaux™ (2400 mg/d) for 28 d each intervention, with a 14 d washout period between each treatment phase. ACTAZIN™ L (low dose, green kiwifruit) and ACTAZIN™ H (high dose, green kiwifruit) were formulated from cold-processed *Actinidia chinensis* var. *deliciosa* ‘Hayward’ green kiwifruit and Livaux™ was formulated from cold-processed *Actinidia chinensis* var. *chinensis* ‘Zesy002’ gold-fleshed kiwifruit. The placebo ingredient was isomalt (1-*O*-α-d-glucopyranosyl-d-mannitol). Subjects were asked to exclude high-fibre dietary supplements such as Metamucil, Benefibre and Phloe as well as maintaining their habitual dietary intakes and physical activity habits and to refrain from eating fresh kiwifruit for the study period. At the beginning and end of each 4-week intervention period, participants were asked to provide a faecal sample. The washout period of 2 weeks was chosen to allow sufficient time to return bowel habits to baseline for the parameters measured (microbial ecology, microbial metabolites and SmartPill^®^ pH measurements). This study was conducted according to the guidelines laid down in the Declaration of Helsinki and all procedures involving human subjects/patients were approved by the New Zealand Human Disability and Ethics Committee (application number 12/STH/72/AM01). Written informed consent was obtained from all subjects/patients. The trial was registered with the Australia New Zealand Clinical Trials Registry (registration number ACTRN: 12612001270808) (http://www.anzctr.org.au/).

**Table 1.** Description of intervention composition

Intervention	Treatment	Dose (mg)	Delivery – capsules/d
ACTAZIN™ L	Green kiwifruit powder	600	1 × ACTAZIN™ + 3 × placebo
ACTAZIN™ H	Green kiwifruit powder	2400	4 × ACTAZIN™
Livaux™	Gold kiwifruit powder	2400	4 × Livaux™
Placebo	Isomalt coloured green (E102, E142)	2400	4 × placebo

L, low dose; H, high dose.

### DNA extraction and 16S rRNA gene sequencing

All faecal samples were collected and stored at −20°C until analysis. Each sample (250 mg) was weighed into a sterile microtube and DNA extracted using the MO-BIO PowerSoil^®^ DNA Isolation Kit (catalogue no. 12888; MO-BIO Laboratories). Illumina MiSeq 16S rRNA gene sequencing was performed as described in a previous study^(^^16^^)^.

### Bioinformatics

Quantitative Insights Into Microbial Ecology (QIIME) software version 1.8.0 was used to analyse the Illumina MiSeq sequencing data^(^^17^^)^. To assemble paired-end reads into a single continuous sequence, PANDASeq was used with parameters of at least 40 bp overlap, a minimum of 350 bp length and maximum of 500 bp length^(^^18^^)^. Putative chimeras were filtered from the sequences and the reads clustered into operational taxonomic units based on a 97 % identity threshold value using USEARCH and UCLUST^(^^19^^)^. A subsample of the total reads was taken to allow faster processing of the samples and to normalise at approximately 15 000 reads per sample, which is sufficient for phylogenetic and taxonomic assignment^(^^20^^,^^21^^)^. Alignment of the sequences was carried out using PyNAST^(^^22^^)^ with reference to the Greengenes core reference database (version 13_8)^(^^23^^)^. Taxonomic assignment was made using the Ribosomal Database Project (RDP) naive Bayesian classifier^(^^24^^)^. Healthy and functionally constipated (FC) groups were analysed separately and the effect of each of the four treatments on microbial community composition determined by comparing the average abundance of each bacterial genus following treatment (greater than 1 % abundance in at least one of the eight samples) with the average value before intervention.

### Organic acid quantification by GC

A 500–1000 mg portion of each faecal sample was weighed into a clean tube and diluted 1:10 in PBS. An internal standard (ethyl butyrate) was included to give a final concentration of 5 mm. Organic acids were quantified by GC using a modified method^(^^25^^)^. Analysis was performed on a Shimadzu GC system (GC-17A) equipped with a flame ionisation detector and fitted with an HP-1 column (Agilent Technologies). The instrument was controlled and chromatograms acquired using GC Solution Chromatography Data System software (Shimadzu, version 2.3). Organic acid concentrations were expressed as μmol/g faeces.

### SmartPill®

SmartPill^®^ is a single-use, ingestible capsule and used in clinical medicine as an alternative approach for determining gastrointestinal transit time^(^^26^^)^ instead of using radio-opaque markers and scintigraphy. SmartPill^®^ is able to measure *in situ* gastrointestinal pH, pressure and temperature and uses telemetry to relay information wirelessly to a small receiving device worn during the study. Six participants from the FC group underwent SmartPill^®^ testing whilst on either the placebo or ACTAZIN™ H treatments. SmartPill^®^ data were compared between treatments to ascertain whether ingestion of ACTAZIN™ H capsules had an impact on pH. Migration of the SmartPill^®^ capsule past the ileocaecal junction into the proximal colon was characterised by a distinct decline in pH. The entire colonic pH from the ileocaecal junction to passing the capsule was averaged and compared between treatments for all participants. Faecal samples originate from the distal bowel, therefore distal rectosigmoid colon pH was determined by taking the last 35·4 % of total colonic pH measurements as calculated in a study interpreting segmental colonic transit times^(^^27^^)^. Bacterial groups were correlated with distal colonic pH data from SmartPill^®^ measurements. A graphical example of SmartPill^®^ data output for a participant is shown in Supplementary Fig. S2.

### Statistical analysis

Statistical calculations were conducted in RStudio using the statistics package^(^^28^^)^. Bacterial groups were correlated with rectosigmoid pH data from SmartPill^®^ measurements using the Spearman's rank correlation calculation. The Wilcoxon signed rank test was performed to assess significant differences between taxa and significant differences between organic acid concentrations before and after each treatment. A *P* value of less than 0·05 was deemed significant after correcting for multiple comparisons using the false discovery rate (FDR) method in the p.adjust function in RStudio^(^^29^^)^.

## Results

### 16S rRNA gene sequencing

High-throughput sequencing of variable regions of the 16S rRNA gene, amplified from faecal sample-derived bacterial DNA, resulted in 26·3 million reads. After quality filtering, chimera removal and subsampling, a total of 3·72 million reads were obtained at an average of 14 879 (14 139 minimum–14 999 maximum) sequences per sample. Over all samples, 218 species-level phylotypes were observed at a 97 % sequence identity threshold.

**Fig. 1.**
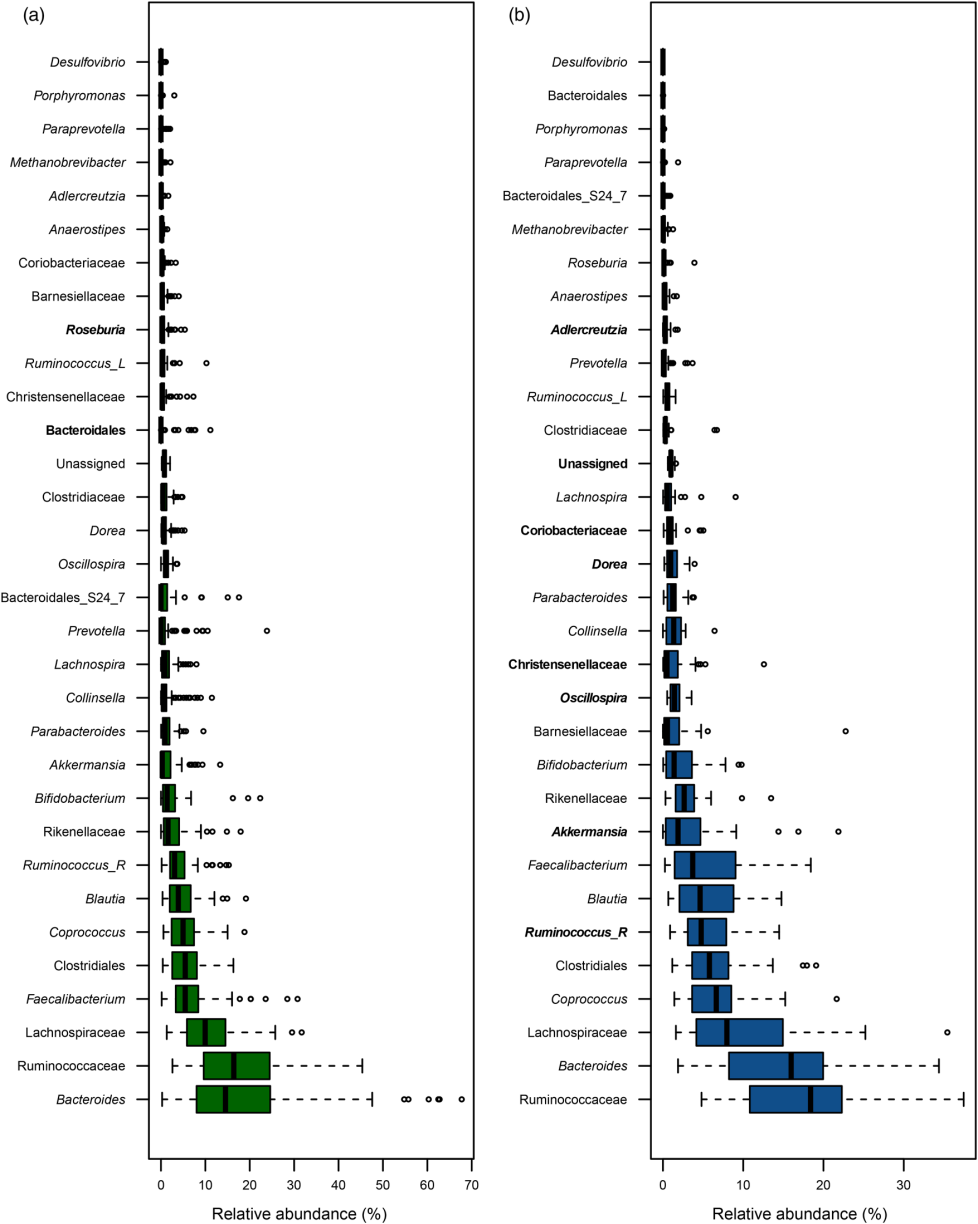
Boxplots displaying bacterial taxa in healthy (a) and functionally constipated (b) baseline samples of faecal microbiota that are present at greater than 1 % abundance in at least one sample. The genera in bold font are significantly more abundant compared with the other group, as calculated by the Wilcoxon signed rank test after false discovery rate correction for multiple comparisons (*P* ≤ 0·05). *Ruminococcus_R*, from Ruminococcaceae family; *Ruminococcus_L*, from Lachnospiraceae family.

Analysis of the participants’ faecal microbiota composition before intervention showed marked differences between the healthy and FC cohorts. A total of thirty-two genera were measured at greater than 1 % abundance in at least one sample, ten of which differed significantly between the baseline healthy and FC groups (Fig. 1). Furthermore, the ratio of Firmicutes to Bacteroidetes differed between the groups at 2·25 and 3·19 for the healthy and FC groups, respectively. These numbers agree with other studies that show an increase in Firmicutes in irritable bowel syndrome patients relative to healthy controls^(^^30^^)^.

In the healthy group, Clostridiales increased significantly after Livaux™ supplementation from 5·0 to 7·6 % (*P* = 0·042) (Table 2). In the FC group, *Dorea* spp. increased from 0·9 to 1·4 % (*P* = 0·008) after the ACTAZIN™ H treatment (Table 3). In the FC group, *Faecalibacterium prausnitzii* significantly increased after Livaux™ treatment from 3·4 to 7·0 % (*P* = 0·024), a two-fold increase as can be seen in Table 3. Individual participant responses can be seen in Supplementary Fig. S3, where eight out of the nine participants exhibited a net increase in *F. prausnitzii*.

**Table 2.** Relative abundance of prevalent bacterial groups in response to treatments in the healthy group†

	Placebo				Livaux™				ACTAZIN™ L				ACTAZIN™ H			
	Pre		Post		Pre		Post		Pre		Post		Pre		Post	
	Mean	sem	Mean	sem	Mean	sem	Mean	sem	Mean	sem	Mean	sem	Mean	sem	Mean	sem
*Bacteroides*	18·8	4·0	18·0	3·2	23·1	4·6	22·0	4·2	16·7	3·1	18·4	3·9	19·4	3·2	18·1	3·8
Ruminococcaceae‡	16·3	2·4	19·6	3·0	17·4	2·3	17·8	2·5	19·9	2·5	22·2	3·8	18·4	2·6	19·9	3·3
Lachnospiraceae‡	10·8	1·3	11·0	1·5	11·9	1·9	9·5	1·3	11·6	1·6	9·3	1·7	11·6	1·6	11·0	1·4
*Faecalibacterium*	7·0	1·5	5·7	1·0	7·2	1·6	5·3	0·9	6·3	1·6	4·6	0·8	6·7	1·1	6·5	1·6
Clostridiales‡	5·9	0·9	5·7	1·0	5·0	0·9	7·6*	1·1	5·9	0·7	5·0	1·1	6·6	1·2	5·6	0·9
*Coprococcus*	6·6	1·0	5·5	0·8	5·9	1·1	5·1	0·7	5·4	0·8	5·2	0·8	5·1	0·8	4·9	0·8
*Blautia*	5·8	0·9	4·7	1·0	4·3	0·6	4·8	1·0	4·7	0·7	6·3	1·7	4·8	1·1	6·1	1·8
*Ruminococcus_R*	4·4	1·0	5·9	1·6	3·8	0·6	3·7	1·0	5·3	1·0	5·9	1·3	3·5	0·8	4·4	1·2
Rikenellaceae‡	2·8	0·9	2·7	0·7	2·7	0·7	2·9	1·0	2·8	1·0	2·5	0·9	3·0	0·6	3·5	0·8
*Bifidobacterium*	3·0	1·0	2·3	0·6	2·5	1·2	2·1	0·6	2·0	0·4	3·5	1·1	2·7	0·9	2·6	0·8
Bacteroidales‡	2·2	1·0	2·2	0·8	2·0	0·9	1·9	0·8	1·3	0·4	1·2	0·3	2·6	1·1	1·7	0·6
*Lachnospira*	1·6	0·5	1·1	0·2	1·3	0·3	1·5	0·5	1·0	0·2	1·1	0·3	1·8	0·5	1·3	0·4
*Parabacteroides*	1·2	0·3	1·6	0·5	1·8	0·6	1·1	0·2	1·3	0·3	1·0	0·2	1·7	0·3	1·5	0·3
*Collinsella*	1·8	0·7	1·2	0·4	0·9	0·4	1·3	0·4	1·6	0·6	1·2	0·3	1·5	0·6	1·1	0·3
*Akkermansia*	1·3	0·5	1·4	0·6	1·8	0·7	1·6	0·7	1·6	0·6	1·8	0·5	1·4	0·4	1·0	0·3
*Oscillospira*	1·1	0·2	1·1	0·2	1·1	0·2	1·6	0·3	1·4	0·2	1·5	0·5	1·2	0·1	1·5	0·2
*Prevotella*	1·5	0·7	2·6	1·8	0·8	0·4	2·1	1·5	2·5	1·33	0·7	0·3	0·9	0·5	1·6	0·9
*Dorea*	1·2	0·4	1·2	0·3	0·9	0·2	1·5	0·4	1·1	0·2	0·9	0·2	0·8	0·2	1·0	0·2
Clostridiaceae‡	1·5	0·4	0·9	0·3	0·9	0·2	0·9	0·2	0·8	0·2	1·2	0·4	0·8	0·2	0·9	0·3
Unassigned	0·8	0·1	1·0	0·2	0·8	0·1	1·0	0·2	0·9	0·1	0·9	0·1	0·8	0·1	0·8	0·1

L, low dose; H, high dose; *Ruminococcus_R*, from Ruminococcaceae family.

* Significantly different compared with pre-treatment based on the Wilcoxon signed rank test after false discovery rate correction for multiple comparisons (*P* ≤ 0·05).

† Illumina MiSeq sequencing data displaying genera that are present at greater than 1 % abundance in at least one sample. Data are the calculated average values for all participants with their standard errors before and after each treatment period.

‡ Some bacteria could only be classified as far as the order or family level.

**Table 3.** Relative abundance of prevalent bacterial groups in response to treatments in the functionally constipated group†

	Placebo				Livaux™				ACTAZIN™ L				ACTAZIN™ H			
	Pre		Post		Pre		Post		Pre		Post		Pre		Post	
	Mean	sem	Mean	sem	Mean	sem	Mean	sem	Mean	sem	Mean	sem	Mean	sem	Mean	sem
Ruminococcaceae‡	17·2	2·3	19·9	2·9	17·1	3·8	16·3	2·1	17·9	2·6	15·0	2·3	16·5	3·1	18·8	2·1
*Bacteroides*	12·6	3·1	14·4	2·2	14·2	2·6	15·5	2·6	13·9	2·2	12·2	2·5	14·0	2·9	16·3	3·1
Lachnospiraceae‡	11·4	1·6	8·0	2·3	10·0	2·5	13·9	3·3	10·8	3·1	11·0	2·3	11·7	2·5	8·7	2·2
*Faecalibacterium*	7·9	2·8	5·8	2·1	3·4	1·0	7·0*	1·1	4·9	1·7	5·0	1·4	7·7	2·1	3·1	1·0
Clostridiales‡	4·8	0·6	6·8	1·7	6·4	1·3	5·5	1·1	6·4	1·2	6·7	1·7	6·0	1·4	7·5	1·8
*Coprococcus*	6·5	1·4	6·1	1·1	6·7	1·7	6·6	1·4	6·9	1·4	8·6	1·2	6·0	1·2	7·1	1·9
*Blautia*	6·6	1·8	3·5	0·9	7·1	2·0	6·0	1·5	6·9	1·5	7·5	1·8	5·9	2·1	5·4	1·3
*Ruminococcus_R*	9·4	2·6	5·1	1·0	5·7	1·4	4·9	0·7	5·5	2·0	6·7	1·5	5·1	1·1	6·6	1·3
*Bifidobacterium*	2·6	1·3	2·6	1·0	3·5	1·5	2·6	1·0	2·7	1·5	2·7	0·9	4·0	1·6	1·8	0·8
*Akkermansia*	2·7	1·7	4·8	2·0	5·0	1·8	2·6	1·0	3·3	1·1	3·3	1·5	3·6	1·0	5·6	2·3
Rikenellaceae‡	2·3	0·6	3·1	0·6	3·9	1·2	2·4	0·5	4·0	1·1	3·9	1·3	2·6	0·7	3·2	0·9
Christensenellaceae‡	1·0	0·6	1·5	0·6	2·2	1·4	1·1	0·6	1·8	0·8	1·4	0·6	2·3	1·6	2·2	1·3
*Prevotella*	1·8	1·1	0·6	0·3	0·2	0·1	0·5	0·3	0·5	0·3	0·9	0·5	2·0	1·1	0·2	0·1
*Collinsella*	1·6	0·6	1·2	0·3	1·6	0·4	1·3	0·3	1·2	0·4	1·8	0·7	1·6	0·6	1·4	0·3
*Parabacteroides*	1·3	0·4	1·3	0·4	1·2	0·3	1·3	0·4	1·7	0·4	1·2	0·4	1·3	0·4	1·5	0·3
*Oscillospira*	1·3	0·1	1·5	0·3	1·6	0·2	1·8	0·3	1·6	0·2	1·5	0·3	1·3	0·2	1·8	0·3
*Lachnospira*	1·0	0·5	1·0	0·4	0·6	0·2	1·6	0·9	1·3	0·5	1·2	0·5	1·3	0·6	0·5	0·1
*Dorea*	1·5	0·4	1·4	0·3	1·3	0·3	1·2	0·3	1·2	0·3	1·2	0·4	0·9	0·2	1·4*	0·3
Coriobacteriaceae‡	0·9	0·3	1·2	0·5	1·5	0·4	1·2	0·5	1·1	0·3	1·1	0·3	0·8	0·2	1·2	0·4
Barnesiellaceae‡	0·9	0·3	4·0	2·4	1·0	0·4	1·0	0·3	1·1	0·3	1·4	0·5	0·8	0·3	0·8	0·3
Clostridiaceae‡	0·4	0·1	1·5	0·7	0·9	0·5	1·0	0·7	0·3	0·1	0·4	0·1	0·4	0·1	0·3	0·1
Unassigned	1·0	0·1	1·1	0·1	1·1	0·2	1·0	0·1	1·0	0·1	1·0	0·1	1·3	0·4	1·0	0·1

L, low dose; H, high dose; *Ruminococcus_R*, from Ruminococcaceae family.

* Significantly different compared with pre-treatment based on the Wilcoxon signed rank test after false discovery rate correction for multiple comparisons (*P* ≤ 0·05).

† Illumina MiSeq sequencing data displaying genera that are present at greater than 1 % abundance in at least one sample. Data are the calculated average values for all participants with their standard errors before and after each treatment period.

‡ Some bacteria could only be classified as far as the order or family level.

### Organic acid production

**Fig. 2.**
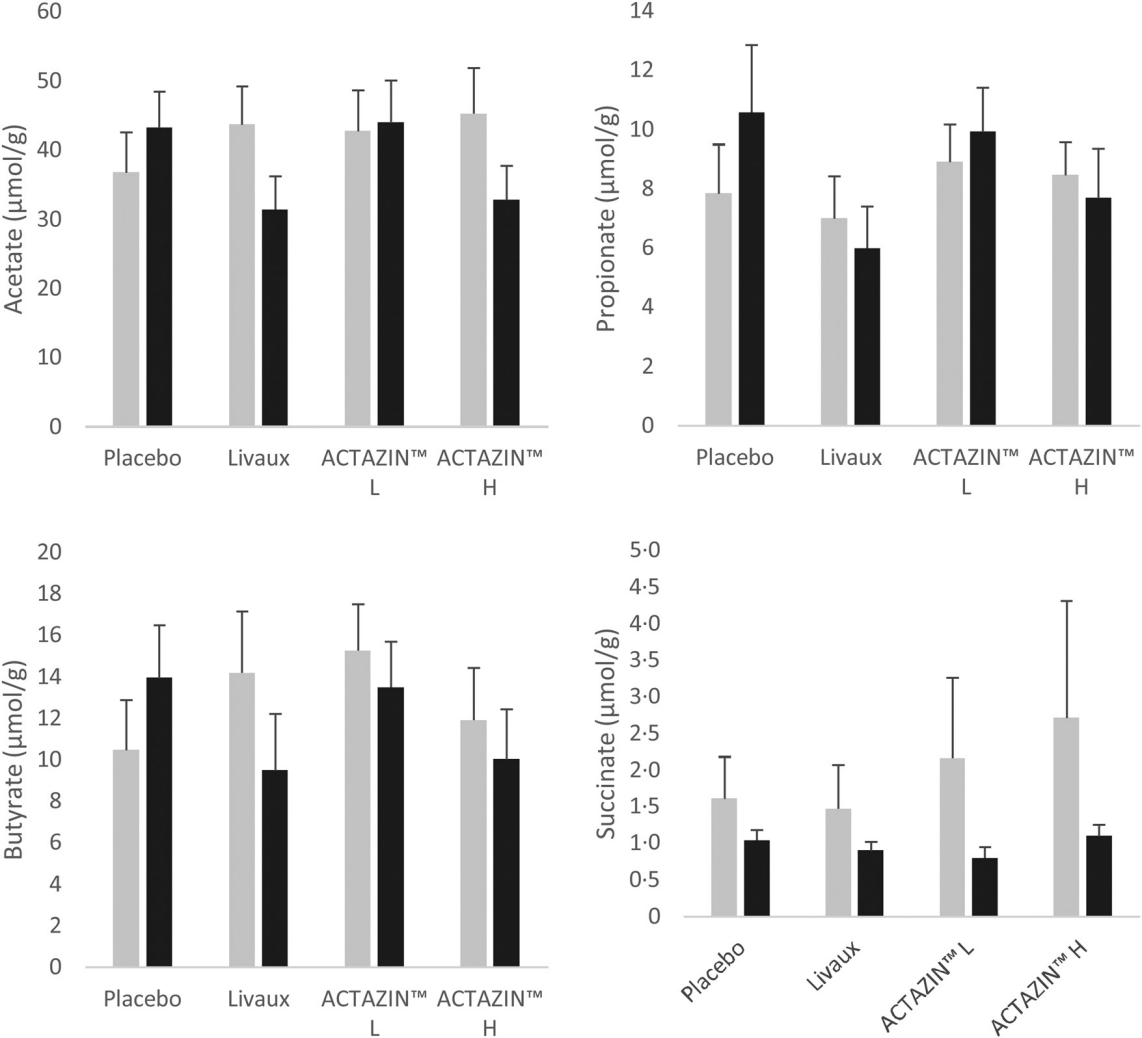
Organic acid concentrations in faecal samples in response to the four treatments in the healthy group as measured by GC, expressed in μmol/g faeces. ░, Pre-treatment; ■, after treatment; L, low dose; H, high dose. Values are means, with standard errors represented by vertical bars.

**Fig. 3.**
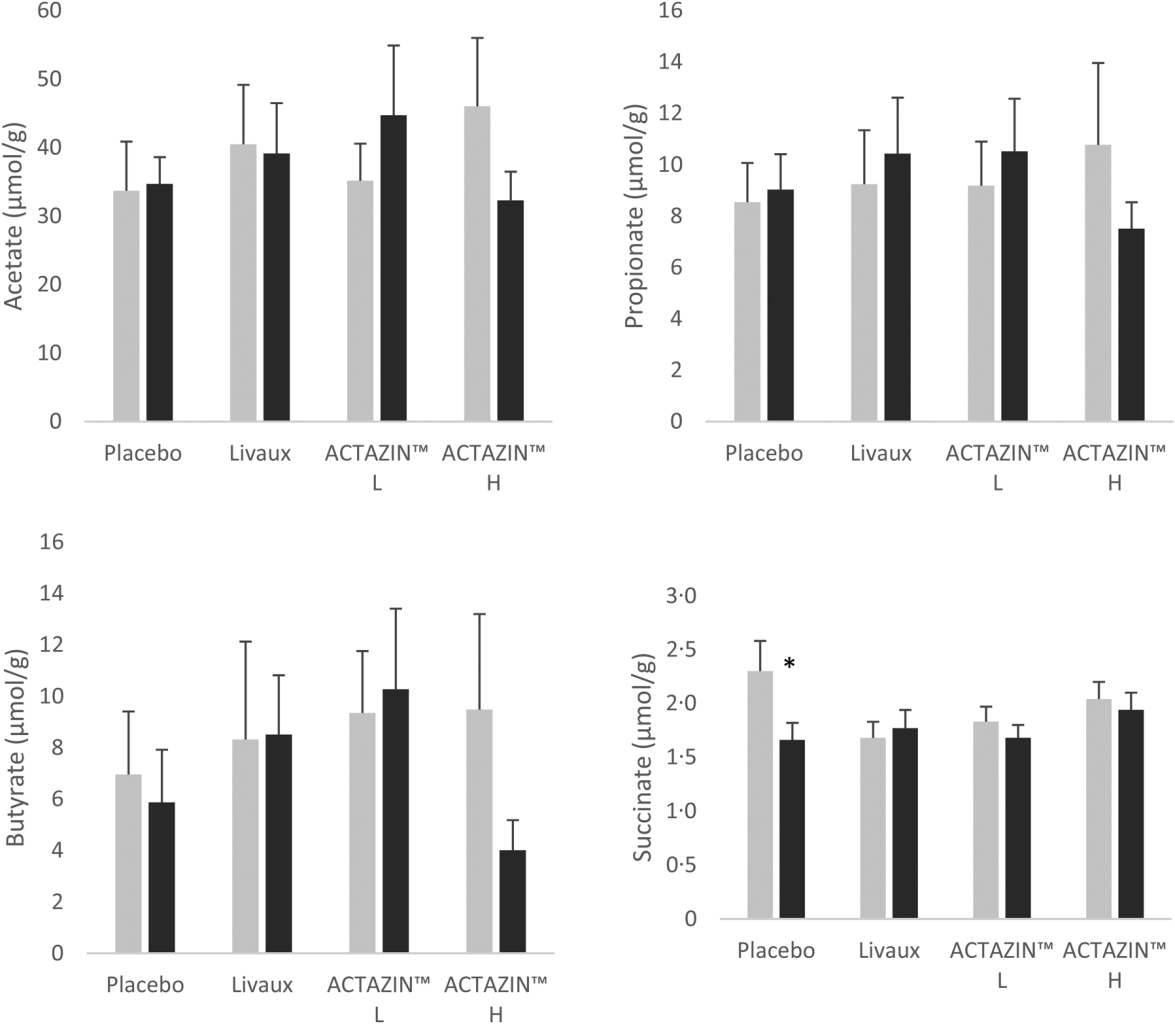
Organic acid concentrations in faecal samples in response to the four treatments in the functionally constipated group as measured by GC, expressed in μmol/g faeces. ░, Pre-treatment; ■, after treatment; L, low dose; H, high dose. Values are means, with standard errors represented by vertical bars. * Significantly different compared with pre-treatment based on the Wilcoxon signed rank test after false discovery rate correction for multiple comparisons (*P* ≤ 0·05).

Succinate significantly decreased in concentration from 2·3 to 1·7 µmol/g (*P* = 0·040) following placebo treatment in the FC group (Fig. 3). There were no other significant alterations to organic acid concentrations with any of the treatments. Quantitative differences before and after each treatment were generally modest, except for acetate which increased or decreased by up to 13 µmol/g after some treatments (Figs 2 and 3).

### SmartPill®

SmartPill^®^ data were used to determine the pH of the colonic environment in a small group of participants taking the ACTAZIN™ H capsules and the placebo treatment. Comparable pH values were observed between treatments, with an average pH of 6·38 in the ACTAZIN H treatment and an average pH of 6·30 in the placebo treatment (Supplementary Table S4). A Spearman rank correlation between bacterial groups and rectosigmoid pH provided two significant values: *Bifidobacterium* spp. negatively correlated with pH (−0·41; *P* = 0·044) and *Coprococcus* spp. positively correlated with pH (0·43; *P* = 0·039).

## Discussion

Other results arising from this trial, assessing laxation endpoints, were published separately and showed that the kiwifruit supplements were able to significantly increase bowel movements in healthy individuals^(^^15^^)^. There is a strong relationship between stool consistency and microbiota composition and diversity^(^^31^^,^^32^^)^, which is unsurprising as microbial metabolic end products such as SCFA and gases can affect colonic transit time^(^^33^^,^^34^^)^. However, in this trial no correlations were observed in those participants that exhibited an increase in stool frequency and microbiota composition. Comparison between the baseline microbiota composition of the healthy and FC groups has provided valuable insight to the bacterial aetiology of functional constipation. Interesting findings between the cohorts were a significantly higher Bacteroidales abundance in healthy individuals and significantly higher *Akkermansia* spp. abundance in FC participants. Both patterns agree with a study assessing microbiota composition and stool consistency^(^^32^^)^.

In the present study, we found that several bacterial groups were significantly altered in abundance following kiwifruit supplementation. Clostridiales increased by 2·6 % after Livaux™ supplementation in the healthy group, but as the order includes a wide range of families and genera, including Lachnospiraceae, Clostridiaceae, Ruminococcaceae, Veillonellaceae, etc. and some pathogens, it is difficult to draw meaningful conclusions about the consequence of this increase. *Dorea* spp. increased modestly after ACTAZIN H treatment in the FC group and these members of the diverse Lachnospiraceae family are major gas producers, mainly CO_2_ and H_2_^(^^35^^)^.

A non-significant decrease in *F. prausnitzii* from 7·2 to 5·3 % was observed in the healthy group which could be due to already high levels at baseline. Other studies have observed depleted *F. prausnitzii* in irritable bowel syndrome patients compared with healthy control subjects^(^^30^^)^. It has been demonstrated that the effect of baseline concentrations of a bacterial group may have a substantial bearing on the magnitude of effect observed in response to a dietary intervention^(^^36^^,^^37^^)^. *F*. *prausnitzii* abundance was significantly elevated (3·6 %) in the FC group after a 4-week period of supplementation with the gold kiwifruit-based Livaux™. *F*. *prausnitzii* is one of the more populous species in the human gastrointestinal tract, being typically observed at over 5 % of the total proportion of the colonic microbiota of healthy adults^(^^38^^)^, in agreement with abundance data presented in this study. Members of the Firmicutes phylum, *F*. *prausnitzii* are commensal inhabitants of the human large bowel, with demonstrated anti-inflammatory properties *in vivo*^(^^39^^,^^40^^)^. One study found that *F. prausnitzii* was able to utilise pectin and uronic acids as substrates for growth and both are known constituents of kiwifruit^(^^41^^)^. Green and gold kiwifruit have been shown to exhibit differences in digestibility, leading to altered soluble and insoluble fibre compositions reaching the large bowel^(^^42^^)^, which may be why differences were observed in the effect of green and gold kiwifruit fibre in this study. Low amounts of *F*. *prausnitzii* have been associated with a range of intestinal disorders including irritable bowel syndrome, atopy, diabetes and inflammatory bowel diseases such as Crohn's disease and ulcerative colitis^(^^30^^,^^39^^,^^40^^,^^43^^,^^44^^)^. These consistent observations show that a depleted concentration of *F. prausnitzii* is an undesirable endpoint and therefore any treatment that can selectively stimulate its proliferation *in situ* is likely to be a promising intervention strategy.

The mechanisms by which *F. prausnitzii* facilitates its health-promoting impact are most probably through butyrate production and subsequent anti-inflammatory effects. Butyrate is the preferred energy source for colonic epithelial cells and plays a role in alleviating inflammation as well as mitigating carcinogenesis, pathogenic colonisation and oxidative stress^(^^12^^,^^45^^)^. A mouse study by Sokol *et al*.^(^^40^^)^ found that *F. prausnitzii* or *F. prausnitzii* culture supernatant reduced the severity of chemical-induced colitis, promoted the synthesis of anti-inflammatory cytokines and mitigated pro-inflammatory cytokine production, suggesting that anti-inflammatory effects are mediated by secreted metabolites. Therefore, increasing the amount of *F. prausnitzii* in the colon may help mitigate the symptoms of gastrointestinal disorders, potentially through elevated butyrate production.

There were minor differences between organic acids after treatments which could be attributed to using faecal samples as a proxy for *in situ* determination of organic acid composition and concentration. Organic acid concentrations decrease distally in the large bowel due to a number of reasons including secondary fermentation, absorption into the bloodstream and/or utilisation of organic acids (particularly butyrate) by colonocytes. Therefore, measurement of organic acids in faecal samples would greatly underestimate the concentration of *in situ* organic acids^(^^46^^)^. Despite these limitations, butyrate was slightly higher in faecal samples from participants after Livaux™ treatment in the FC group, which aligns with the increased *F. prausnitzii* abundance but it was not to a significant level.

It has been demonstrated that pH can alter microbial populations^(^^47^^)^ and therefore it stands to reason that an altered pH in the colon could modify the colonic microbial profile. In the present study, correlating pH with bacterial groups has revealed one significantly positive and one significantly negative correlation. The discovery that *Bifidobacterium* spp. abundance was significantly higher at lower pH is an expected result as it is an acid-producing bacterium, and hence tolerant of such conditions. Increased bifidobacteria abundance is positive because it is recognised as an established probiotic genus, and in general a lower pH is seen as desirable as it can exclude the growth of competing pathogens^(^^48^^)^.

In conclusion, Livaux™ appears to stimulate proliferation of the commensal *F. prausnitzii* in participants with low initial *F. prausnitzii* concentrations and this may be associated with higher *in situ* butyrate concentrations, although this is difficult to measure in a human study. These results bear clinical relevance as stimulating an increase in an abundant butyrogenic bacterium could have beneficial consequences for ulcerative colitis patients. Future work to corroborate these results could entail i*n vitro* studies to measure butyrate production and *F. prausnitzii* stimulation in a closed system.

## Supplementary material

For supplementary material accompanying this paper visit http://dx.doi.org/10.1017/jns.2017.52. click here to view supplementary material
