# Supplementary material for: Consumption of kiwifruit capsules increases Faecalibacterium prausnitzii abundance in functionally constipated individuals: a randomised controlled human trial
Source: J Nutr Sci. 2017 Oct 12;6:e52. doi: 10.1017/jns.2017.52 (PMC5672330; doi:10.1017/jns.2017.52)
Supplement: Supplementary file 1 [file S2048679017000520sup001.pdf]

## Supplementary Figures

**Figure S1.** Schematic of trial treatments, washouts and sampling points.

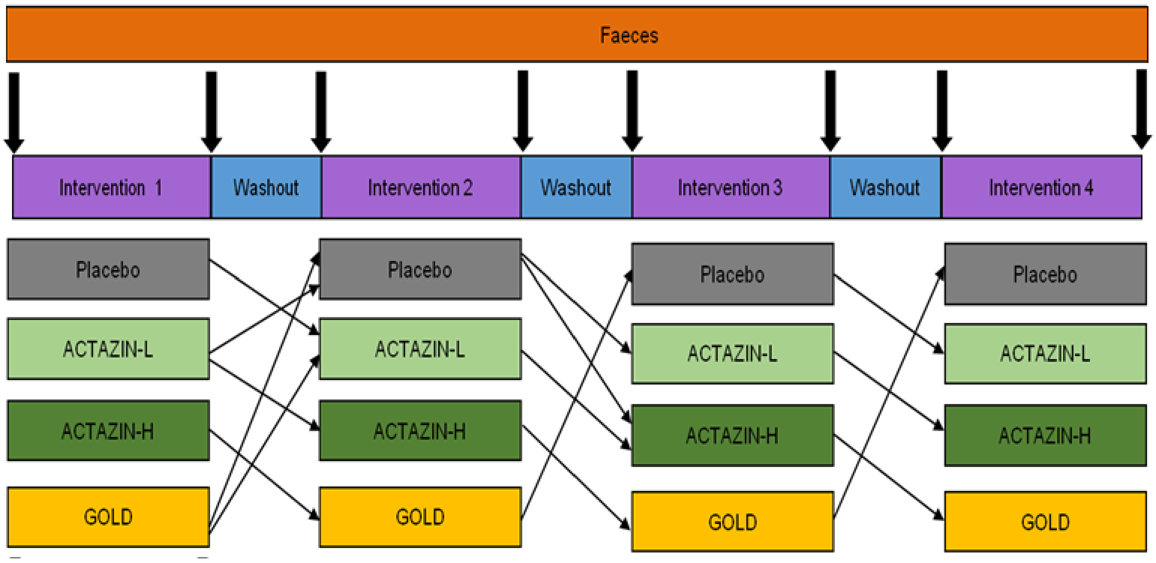


**Figure S2.** SmartPill® data output for a single participant showing pressure (red), temperature (blue) and pH (green) measurements *in situ* during gastrointestinal transit. Transit events (ingestion, gastric emptying, ileocecal junction, exiting the body) are shown (blue, grey, green and pink bars, respectively).

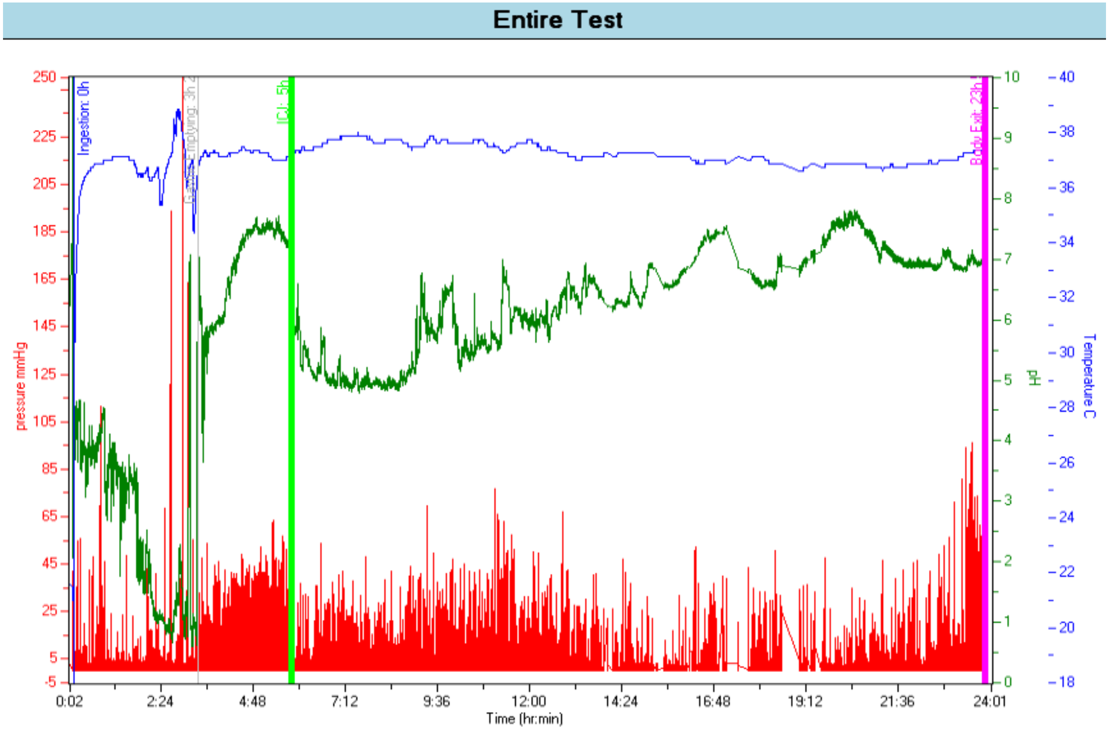

**Figure S3.** Net change in *Faecalibacterium prausnitzii* abundance after Livaux treatment for the nine participants of the functionally constipated group as measured by Illumina MiSeq sequencing.

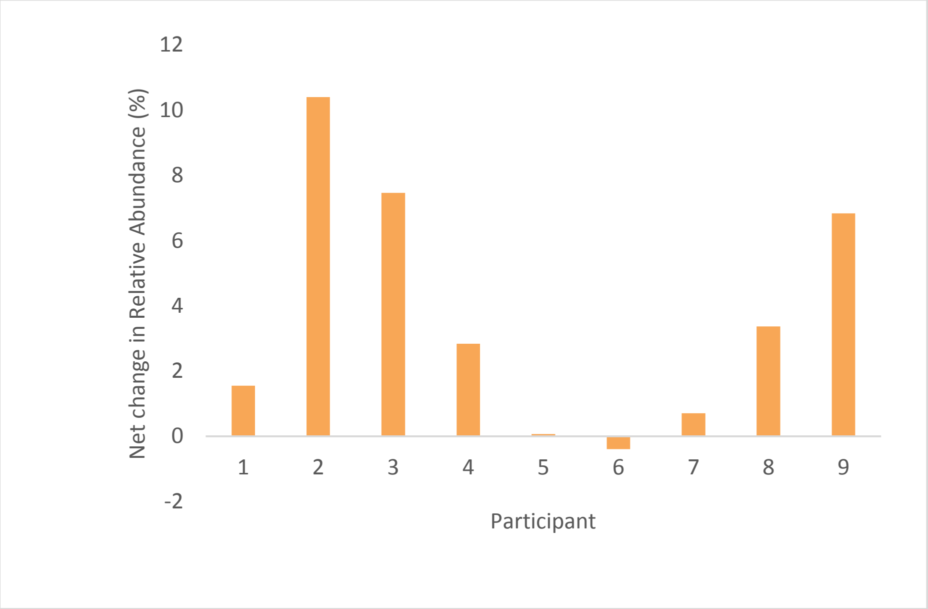


**Table S4.** Average distal colonic pH data from SmartPill® measurements from the six participants while on the placebo or ACTAZIN™ H treatments. Standard error of the mean (SEM).

| | **Placebo** | **Actazin™ H** |
|---|---|---|
| Participant 1 | 7.00 | 6.30 |
| Participant 2 | 6.84 | 6.70 |
| Participant 3 | 6.10 | 6.03 |
| Participant 4 | 6.30 | 5.78 |
| Participant 5 | 5.90 | 6.77 |
| Participant 6 | 5.67 | 6.70 |
| Average | 6.30 | 6.38 |
| SEM | 0.21 | 0.17 |
